# Comparison of the 1 and 2% pilocarpine mouthwash in a xerostomic population: a randomized clinical trial

**DOI:** 10.1186/s12903-022-02576-6

**Published:** 2022-12-01

**Authors:** Babak Motamed, Arezoo Alaee, Arash Azizi, Hoda Jahandar, Mohammad Javad Kharazi Fard, Aryan Jafari

**Affiliations:** 1 Member of Dental Materials Research Center, School of Dentistry, Islamic Azad University - Tehran Medical Branch, Tehran, Iran; 2 Department of Oral Medicine, Member of the dental material research center, School of Dentistry, Islamic Azad University - Tehran Medical Branch, No.9, Neyestan 9, Pasdaran St., Tehran, Iran; 3 Department of Oral Medicine, School of Dentistry, Islamic Azad University Tehran Medical Branch, Tehran, Iran; 4 Pharmaceutical Sciences Research Center, Tehran Medical Sciences, Islamic Azad University, Tehran, Iran; 5 Epidemiologist, Dental Research Center, Dentistry Research Institute, Tehran University of Medical Sciences, Tehran, Iran

**Keywords:** Pilocarpine, Xerostomia, Dry mouth, Oral health, Saliva measurement methods, Muscarinic agonists

## Abstract

**Aims & background:**

Pilocarpine is an accepted treatment for xerostomia, but limited research has been conducted on the oral, topical form. The present study aimed to compare the effects of 1 and 2% pilocarpine mouthwash on xerostomic participants.

**Methods:**

In this double-blind clinical trial study, 48 subjects with xerostomia were randomly divided into three groups to measure the effects of 1 and 2% pilocarpine and placebo mouthwashes on saliva levels. The amount of saliva in the 1st and 14th days was measured at 0, 45, 60, and 75 mins, while participants used their mouthwash three times a day for 14 days. On the 1st and 14th days, they filled out the information forms on xerostomia and the medicine’s side effects before and after the intervention.

**Results:**

On the 1st day, the mean salivary flow at 45, 60, and 75 mins in the 2 and 1% pilocarpine mouthwash were significantly higher than in the placebo mouthwash group (*p* < 0.05). On the 14th day, the mean salivary flow time at 45 mins in the 2% pilocarpine mouthwash group was significantly higher than in the placebo mouthwash group (*p* = 0.007). Furthermore, the mean salivary flow at 60 and 75 mins in the 2% (*p* < 0.001) and 1% pilocarpine mouthwash (*p* = 0.028) was significantly higher than in the placebo group. Moreover, the salivary flow in the 2% pilocarpine mouthwash group was significantly higher than the 1% pilocarpine mouthwash (*p* < 0.05) during these two times. No side effects were observed in any of the subjects.

**Conclusions:**

The study showed that 5 ml of 2 and 1% pilocarpine mouthwash for 2 weeks increased salivary flow in xerostomic participants compared to placebo without any side effects.

## Introduction

Xerostomia is a subjective complaint of individuals accompanied by changes in the quality and quantity of saliva. Generally, the sufferers have symptoms that impact their health and the social and emotional elements of their lives. It can also cause many short-term and long-term complications such as tooth caries, burning mouth sensation, periodontal diseases, dysgeusia, difficulty using dentures, and fungal infections resulting in reduced quality of life [1–5]. In addition, hyposalivation is when the unstimulated total salivary flow rate is less than 0.01 mL/min during awake hours [6].

Moreover, xerostomia can be caused by radiation therapy, medications, systemic conditions, smoking, and aging [2, 7]. It has also been linked to COVID-19 in 45.9% of confirmed SARS-CoV-2 patients, with the majority (76.5%) reporting their first time [8]. Xerostomia affects 5 to 39% of the general population and 17 to 40% of community-dwelling seniors. Between 20 and 70% of institutionalized elderly had it, with postmenopausal women having it the most [9, 10].

Most xerostomia treatments focus on keeping the mouth moist [6]. Symptomatic therapies are also used to increase salivation, such as sugar-free gum, herbal remedies like ginger, water gargling, and systemic parasympathomimetic agents, including pilocarpine, bethanechol, and cevimeline [11–13]. Pilocarpine is a cholinergic parasympathomimetic agonist that interacts with the muscarinic-M3 receptors and can produce smooth muscle contraction and stimulates salivary glands’ function. It is often used as a preventative treatment before starting cervical radiotherapy [14, 15].

Pilocarpine has been Food and Drug Administration (FDA) approved and is administered as a solution of 1 mg/ml, eye drops of 1, 2, and 4%, and tablets of 5 and 7.5 mg. These medicines are used systemically three times a day; their maximum dose is 10 mg/ 3 times daily [16, 17]. Besides, peak plasma concentrations of pilocarpine are attained 75 mins after administration of 5 mg tablets. They are primarily removed in the urine, with a half-life of roughly 45 mins for 5 mg dosages, depending on pilocarpine esterase activity [18, 19].

After oral administration of pilocarpine, some people may experience side effects such as tachycardia, sweating, and flushing, which may explain its confined systemic intake [11, 20–22]. Orally administered pilocarpine is contraindicated in individuals with stomach ulcers and uncontrolled asthma. Furthermore, the possibility of cardiovascular consequences associated with systemic delivery is also a consideration [20]. Moreover, pilocarpine’s systemic absorption should be minimized to avoid quick removal metabolization and optimize the region of interest exposure. Therefore, if the beneficial effect of the above medicine can be used topically, in increasing saliva flow and with fewer side effects, it is very desirable to relieve xerostomia [20, 23].

Pereira et al. claimed that topical use of pilocarpine spray of 1.45% did not affect the total amount of stimulated saliva [23]. However, the study by Watanabe et al. stated that pilocarpine oral solution had a rapid effect on reducing xerostomia [24]. The Park study also reported the effectiveness of both 2% mouthwash and 5 mg pilocarpine tablets in relieving dry mouth [25].

Mouthwash on healthy individuals without xerostomia increased salivary flow more effectively than tablets. However, it had a shorter impact due to its effect on minor salivary glands. Comparisons between 1 and 2% pilocarpine on healthy individuals were performed in limited studies and reported conflicting results [20]. Therefore, the current study evaluated the impact of 1 and 2% concentrations of pilocarpine mouthwash on volunteers with xerostomia.

## Materials & methods

The study was performed as a double-blind, parallel, randomized controlled clinical trial on volunteers with xerostomia referred to the School of Dentistry of Tehran Islamic Azad University of Medical Sciences in 2020. Purpose-based sampling was conducted among male and female volunteers aged 18 to 60 who were literate and suffering from xerostomia. Xerostomia was diagnosed when volunteers responded yes to four questions of the Fox questionnaire [26, 27]. The questions were as follows; 1) “Does the amount of saliva in your mouth seem to be too little, too much, or you do not notice it?” 2) “Do you have any difficulty swallowing?” 3) “Does your mouth feel dry when eating a meal?” 4) “Do you sip liquids to aid in swallowing dry food?” They were also asked about their problem’s severity regarding each question according to VAS (visual analog scale) from 1 to 10 and then converted to 0.1–1.

In addition, the exclusion criteria of the sampling included: 1) present pregnancy or lactation, 2) presence of an oral lesion, 3) infection or burning mouth, 4) use of prosthetic dentures, 5) history of previous contact dermatitis or allergic reaction caused by pilocarpine, connective tissue or systemic diseases affecting salivary gland function like Sjögren’s syndrome, Rheumatoid arthritis, systemic lupus erythematosus, progressive systemic sclerosis (PSS), 6) history of head and neck radiotherapy, 7) acquired immunodeficiency syndrome (AIDS), 8) hepatitis B or C, 9) hypertension (above 140/90 mmHg), cardiac or renal diseases, 10) Parkinson’s disease, 11) asthma, 12) chronic obstructive pulmonary disease (COPD), 13) glaucoma, 14) chemical or herbal medication and salivary enhancers intake, and 15) alcohol and tobacco products except for cigarettes [20, 25].

The present study’s protocol followed the Declaration of Helsinki. The ethics committee of the Islamic Azad University Faculty of Dentistry, Tehran, Iran (IR.IAU.DENTAL.REC.1399.282) also approved it on 03/03/2021, and it was subscribed to the Iranian registry of clinical trials (IRCT20210208050302N1). After explaining the steps to the volunteers, all eligible volunteers were asked to complete and sign the informed consent form to participate in the study. The participants were then asked to fill carefully in the demographic information form and the standard xerostomia questionnaire [27] to determine the individual’s initial xerostomia severity [26, 27].

Additionally, using PASS 15 software considering β = 0.2, α = 0.05, and a standard deviation of 0.85 to achieve the effect size of 0.52, the minimum sample size for each group was 16 [24]. The recruitment of volunteers took place from April 2021 to October 2021. After that, the 48 individuals were divided in a 1:1:1 ratio into three groups of 16 by simple randomization by Arezoo Alaee. The groups were almost homogeneous regarding age, gender, smoking, systemic disease, and educational status [20, 24].

**Fig. 1:**
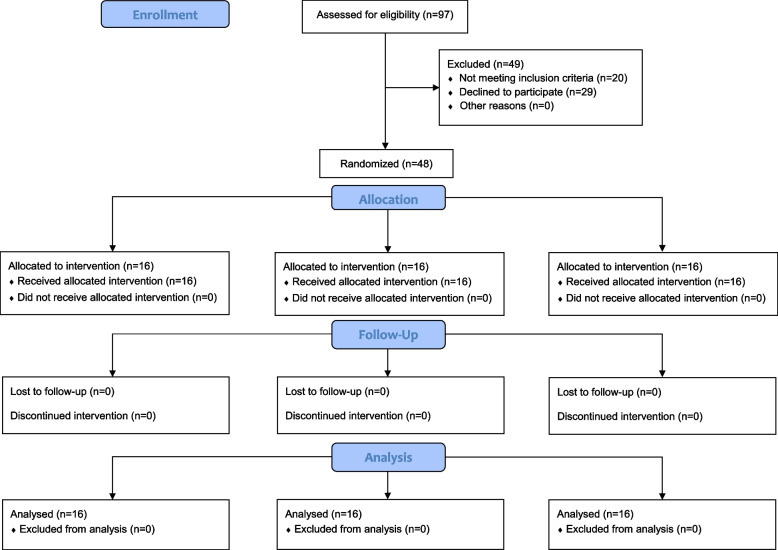
Consolidated Standards of Reporting Trials (CONSORT) diagram of participants’ flow

As demonstrated in the CONSORT flow diagram (Fig. 1), three types of medicinal intervention were administered:

1. Group A: 2% pilocarpine mouthwash, three times a day,
2. Group B: 1% pilocarpine mouthwash, three times a day, and
3. Group C: Placebo mouthwash, three times a day.

Since pilocarpine 2 and 1% mouthwash was not available in the Iranian pharmaceutical market, it was prepared in the laboratory of the Pharmaceutical Sciences Research Center of the Faculty of Pharmacy, the Islamic Azad University of Medical Sciences, Tehran, Iran. The 4% pilocarpine eye drops (Glaupin® 4%, Sina Darou, Iran) were diluted with Vi-one junior mouthwash (Rojin Cosmetic, Iran) to prepare 2% pilocarpine mouthwash and improve the taste and acceptance of participants. Vi-one junior mouthwash is a flavored, alcohol-free product specially designed for kids that contains harmless sweeteners, disodium phosphate agents, 0.05% sodium fluoride, and 0.05% cetylpyridinium chloride [28]. Moreover, the 2% pilocarpine mouthwash was diluted with water to make 1% pilocarpine mouthwash. Furthermore, Vi-One junior mouthwash (Rojin Cosmetic, Iran) was diluted with water to create a similar taste and color as other mouthwashes for the placebo group.

It is worth noting that the pharmacologist author of the current study (Hoda Jahandar) coded the three types of mouthwashes and packaged them like each other. Hence, the participants and the researchers were unaware of the medicines in the package. Besides, high-performance liquid chromatography (HPLC) was utilized (SCL-10AVP, Shimadzu, Japan) to evaluate the stability of this product during the study period (14 days). It was considered that maintaining at least 90% of the area below the pilocarpine curve relative to day zero indicates product stability.

In addition, blood pressure (mmHg) and pulse rate (pulse per second) were recorded on the 1st and 14th day before and 75 mins after medicine application by a hand-held sphygmomanometer (KaWe, Germany). The participants’ weights were also measured with minimum clothing and without shoes with a ± 0.5 kg accuracy. A participant stood next to the wall without shoes, legs paired to each other, head leaning against the wall, to determine their height. Furthermore, body mass index (BMI) was obtained by dividing body weight (kg) by the square of a person’s height (m^2^) [16].

On the 1st day, the amount of unstimulated saliva was measured before mouthwash administration and 45, 60, and 75 mins after gargling the mouthwash for all three groups. The unstimulated saliva was measured by absorption or swab-based technique for participants before intervention. Additionally, the participants were asked to avoid eating, drinking, smoking, and brushing their teeth for at least 2 hrs before accurately measuring the amount of non-stimulated saliva. After rinsing their mouth with 15 ml of distilled water, the participants rested in a room with adequate comfort and ventilation for one min and sat upright on a dental chair.

Afterward, the volunteers in all three groups were asked to gently gargle 5 ml of their mouthwash solution for 1 min without swallowing the contents and then drain it completely. Then, observing the complete principles of sterilization and wearing a mask and face shield, the researcher (Babak Motamed) placed the previously weighed dry cotton roll (1 × 4 cm^2^) in the participant’s mouth. After 1 min, the researcher removed the wet cotton roll with dental pliers, placed it in a coded bag, and sent it to the laboratory for further weighing using a digital scale with ±0.01 g accuracy.

Saliva content (cc or gr/ml) = wet cotton roll weight - dry cotton roll weight.

All salivary measurements were carried out between 9 and 11 AM [29] as saliva secretion may fluctuate during the day. Saliva samples were kept at 4 °C to prevent further changes. The participants’ medication-related side effects were recorded on the first day using an 11-item questionnaire based on the VAS (visual analog scales) before the study [24, 30]. These 11 side-effects were 1) visual difficulties or blurred vision, 2) tremors, 3) indigestion and heartburn, 4) diaphoresis, 5) tachycardia, 6) anxiety, 7) headache, 8) hot flashes, 9) epiphora, 10) sialorrhea and 11) urinary frequency. The xerostomia questionnaire was also filled out again on the 14th day for all three groups of participants. Furthermore, saliva was measured on the 14th day at 0, 45, 60, and 75 mins. On the 14th day, possible side effects of the volunteers based on VAS were also recorded at 0 and 75 mins.

Besides, during these 14 days, the participants had constant contact with the researchers through phone calls to find answers to their questions or possible problems. The participants were also barred from taking medicines that relieve dry mouth or increase saliva. However, water intake, chewing gum, and topical local anesthesia agents were unimpeded.

### Statistical analysis

SPSS software version 26 was used for data analysis. Moreover, Kolmogorov–Smirnov test was used to determine the normality of the data. Chi-square, one-way ANOVA, repeated-measures ANOVA, Tukey, and Independent Samples T-Test were also employed to analyze the data. In addition, values less than 0.05 were considered statistically significant.

## Results

As shown in Table 1, there was no significant difference between the studied groups in gender, mean age, height, weight, and the number of cigarettes smoked per day.

**Table 1:** Demographic characteristics of the studied subjects

Characteristic	Mouthwashes			Total	***P***- value
	2% pilocarpine mouthwash	1% pilocarpine mouthwash	Placebo mouthwash		
**Gender (male)**	7 (43.8%)	8 (50%)	7 (43.8%)	22 (45.8%)	0.92
**Age (year)**	48.00 ± 8.29	49.94 ± 6.68	47.63 ± 8.27	47.63 ± 8.27	0.669
**Height (cm)**	170.38 ± 8.65	168.06 ± 10.03	169.25 ± 9.83	169.25 ± 9.83	0.791
**Weight (kg)**	70.81 ± 7.94	72.06 ± 9.42	69.81 ± 12.46	69.81 ± 12.46	0.821
**Cigarettes per day**	10.19 ± 9.07	8.13 ± 10.15	10.94 ± 10.68	10.94 ± 10.68	0.714

The HPLC results revealed that the sample level below the graph and the symmetry of the peaks in the samples containing pilocarpine 1 and 2% during the study did not change significantly. Moreover, they were in the 90 to 110 range compared to the level below the first day’s graph. Therefore, this product was stable during the study period.

### The salivary flow

No subject was lost to follow-up during the study. Besides, the salivary flow of the three groups and the pairwise comparison of salivary flow before and after the intervention at different times is presented in Table 2. On the 1st and 14th days, the mean salivary flow in the 2 and 1% pilocarpine mouthwash groups significantly increased from 0 mins to 75 mins (*p* < 0.05), while the mean salivary flow in the placebo group did not change significantly during the same time (*p* > 0.05). Furthermore, the mean salivary flow was not significantly different between the 1st and the 14th day at 0, 45, 60, and 75 mins in all of the three groups (*p* > 0.05).

**Table 2:** Salivary flow (mean ± SD) before and after the intervention at different times

Assessment time	Time (mins)	Salivary Flow (ml/min)			***P*** value
		2% pilocarpine mouthwash	1% pilocarpine mouthwash	Placebo mouthwash	
1st day	0	0.33 ± 0.10	0.33 ± 0.10	0.33 ± 0.10	**1.00**
	45	0.57 ± 0.26^a^	0.58 ± 0.22^a^	0.33 ± 0.09	< 0.01*
	60	0.76 ± 0.28^a,b^	0.57 ± 0.19^a,b^	0.35 ± 0.08	< 0.01*
	75	0.72‌ ± 0.26^a,b^	0.51 ± 0.15^a,b^	0.33 ± 0.09	< 0.01*
14th day	0	0.35 ± 0.10	0.34 ± 0.08	0.34 ± 0.09	**0.93**
	45	0.64 ± 0.25^a^	0.56 ± 0.30	0.37 ± 0.14	< 0.01*
	60	0.81 ± 0.21^a,b^	0.54 ± 0.26^a,b^	0.35 ± 0.12	< 0.01*
	75	0.73 ± 0.21^a,b^	0.50 ± 0.23^b^	0.38 ± 0.18	< 0.01*

*Statistically significant; ^a^significant difference with placebo; ^b^ significant difference between pilocarpine mouthwashes;

Moreover, Table 3 compares the xerostomia severity before and after the intervention based on VAS 4-question xerostomia questionnaire results. No significant differences were found in individuals statements before and after treatment.

**Table 3:** Comparison of xerostomia before and after the intervention based on xerostomia questionnaire score according to visual analogue scale (mean ± SD)

Intervention	1st day	14th day	***P*** value
2% pilocarpine mouthwash	2.83 ± 1.60	1.84 ± 1.47	**0.08**
1% pilocarpine mouthwash	2.91 ± 1.16	2.34‌ ± 0.97	**0.15**
Placebo mouthwash	3.09 ± 1.34	2.85 ± 1.12	**0.58**
***P*** **value**	**0.85**	**0.07**	

### Side effects

Based on the side effects questionnaire, there was no significant difference between different groups and at different times (*p* > 0.05). On the 1st and the 14th day, the mean pulse, systolic, and diastolic blood pressure of the participating volunteers at 0 mins and 75 mins were not significantly different between the three groups (*p* > 0.05). Moreover, the mean pulse of the participating subjects at 0 mins and 75 mins was not significantly different between the three groups (*p* > 0.05).

In addition, none of the participants were found to have chewing problems, swallowing problems, taste problems, speech problems, and a burning sensation in the mouth. Furthermore, the participants did not report any of the 11 side effects included in the side-effects questionnaire.

## Discussion

The present study showed that pilocarpine mouthwash significantly increased the salivary flow in xerostomic participants (aged 18–60) at 45, 60, and 75 mins. Moreover, the highest mean salivary flow rate was observed in the 2% pilocarpine mouthwash and 1% pilocarpine mouthwash groups, respectively. It should be noted that systemic intake of pilocarpine tablets has side effects such as sweating, flushing, and increased frequency of urination; thus, there are limitations to its use [17, 20]. Therefore, the mouthwash form was evaluated considering these side effects.

In the present study, topical application of pilocarpine mouthwash in the highest concentration (2%) did not cause a burning sensation in the mouth, increase in blood pressure or pulse rate, nor caused chewing, swallowing, speeching, or taste problems in any of the studied groups. In addition, in none of the studied groups, possible medical side effects were observed compared to the placebo group. The study populations’ responses to the side-effect questionnaire and the recorded heart rates and blood pressures indicated insignificant systemic absorption of pilocarpine. It is believed that the slow absorption of the local solution may be associated with its rare side effects [25]. However, note that some side effects are commonly reported after chronic use of oral pilocarpine and are dose-dependent. Frequent long-term use of topical pilocarpine may cause side effects [17].

No significant differences were found in volunteers' statements before and after treatment. Because the reversal of the atrophic and dry changes due to lack of saliva does not occur immediately after pilocarpine administration, several weeks must elapse before symptomatic relief becomes apparent [20].

Topical and systemic pilocarpine effects on salivation have been reported in individuals with hyposalivation [23]. Recently, a non-placebo-controlled clinical trial pilot study was conducted on ten xerostomic subjects who were prescribed to take either low-dose pilocarpine (3 × 2 mg/day) or high-dose pilocarpine (3 × 5 mg/day). After a week, all high-dose and 57% of low-dose participants reported relief from xerostomia. However, the difference between the two groups was not statistically significant [31]. Additionally, Park et al. [25] found that 2% pilocarpine mouthwash had the same effect on saliva flow as 5 mg pilocarpine tablets when administered systemically to 12 healthy individuals. Consequently, saliva flow increased significantly using pilocarpine tablets and pilocarpine solution. Though the dose of pilocarpine mouthwash used in these studies differed from that of the current study, the findings were similar.

Topical pilocarpine mouthwashes have several advantages, including prolonged contact with the site of action and the mechanical stimulation of salivary glands resulting in mucin production, which has an essential role in symptom salvation [25, 32]. The mechanisms by which pilocarpine affects salivary flow include local and direct cellular stimulation. The parasympathetic effect of pilocarpine causes the flow of water and electrolytes in saliva [20, 33]. Moreover, when using a therapeutic mouthwash, the subject should be instructed to gargle robustly to make the most of mechanical stimulation of the salivary glands [34].

Previous studies have examined the effects of pilocarpine mouthwash at concentrations ranging from 0.01 to 4% and have indicated that the effect increases with concentration [1, 17, 23, 25, 34]. Bernardi’s study examined the effects of 0.5, 1, and 2% pilocarpine mouthwashes in healthy volunteers and showed a dose-dependent increase in whole unstimulated saliva [20]. However, the sample size was small, and the long-term effect was not assessed. Previous studies have shown that pilocarpine solutions at concentrations less than 1% cannot significantly increase salivary flow but can improve subjective symptoms of dry mouth [25]. However, lower concentrations may be helpful in xerostomic subjects without a considerable decrease in salivary secretion [25].

The current study’s findings contrasted with Pereira et al. [23], who applied 1.5% pilocarpine spray topically for 3 months, rested for 1 month, and then reapplied placebo spray for 3 months. The total amount of stimulated saliva was not significantly different between pilocarpine spray and placebo in 40 head and neck cancer patients. However, a slight increase in salivary flow was observed in individuals after the second month, which the researchers attributed to the subject’s unique profiles. These findings differed from the present study for the differences in the study population and how pilocarpine was used.

Pilocarpine may have different effects on xerostomia caused by different etiologies. We considered salivary gland secretory reserve capacity to be more critical than etiology in assessing the response to pilocarpine mouthwash, as previously mentioned by Kim et al. [34] Various studies have had different approaches in subject recruitment, which may affect their outcomes. The study populations of Song et al. and Park et al. were healthy subjects [17, 25], whereas Pereira et al. and Nikles et al. studies were conducted on head & neck cancer patients with less remaining functional salivary gland tissue [23, 33]. The positive effects of pilocarpine mouthwash in young, relatively healthy volunteers obtained in this study cannot guarantee the same results in individuals with severe dry mouths as a consequence of systemic diseases [17].

Xerostomia usually occurs in elderly subjects with one or more systemic diseases. Hence, polypharmacy in these individuals may limit systemic intake of pilocarpine due to possible drug interactions [25]. The wide range of exclusion criteria of our study excluded volunteers with underlying systemic diseases and limited the xerostomia etiology in our study population to smoking, excessive caffeine or spicy food intake, and mental stress [35]. Future studies are encouraged to conduct trials on populations with xerostomia because of systemic diseases.

Different methods are used to measure salivary flow, making it difficult to standardize results. We used a swab-based technique to measure unstimulated saliva, while Kim et al. [34] used two methods for collecting saliva: non-stimulatory spitting collected the whole saliva, and a sialopaper strip collected the salivary glands’ saliva.

The latency time of increased salivation when pilocarpine is administered orally is 15 mins, with a maximum of 60 mins [17, 20]. It is impossible to compare the latency of saliva secretion after oral administration with topical administration of pilocarpine. However, it is clear that after topical administration, the maximum effect should have occurred earlier than 1 hr. and be stable for at least 75 mins. The current study observed the salivary flow increase 45 mins after using the mouthwash, which persisted for 75 mins. This finding was consistent with Bernardi et al. [20] and Song et al. [17]

Unfortunately, to this date, Pilocarpine mouthwashes and tablets are not available in the Iranian pharmaceutical market for xerostomia patients seeking treatment. Apart from artificial saliva, patients’ medications are just limited to the use of pilocarpine eye drops as treatment, which the bitter taste shrinks patients’ cooperation. Since pilocarpine mouthwash has a bitter taste, we used flavored mouthwash as the diluent solution for the placebo control instead of distilled water, as it may mask the bitter taste. We believe that this simple formulation of 1 and 2% pilocarpine mouthwashes will have promising effects on xerostomia symptom alleviation in Iran and other countries with similar shortage.

In addition, the first limitation of the present study was that the results cannot be applied to a wide range of subjects due to the limited subject conditions in this study. Other limitations included a lack of volunteers due to the coronavirus outbreak, difficulty preparing pilocarpine drops in the pharmaceutical market, and struggle following study subjects. Despite the high prevalence of xerostomia, gatekeeping by relatives and friends posed challenges during sample recruitment, as Theunissen et al. [31] previously stated.

## Conclusions

The current clinical study showed that using 5 ml of pilocarpine 2 and 1% mouthwash for 2 weeks increased salivary flow in xerostomic subjects compared to placebo without any side effects. This increase was also concentration-dependent as the impact of 2% pilocarpine mouthwash was more than 1% mouthwash.

## Data Availability

The data supporting this study’s findings are available from the corresponding author (Arezoo Alaee) upon your request.

## References

[1] Sarideechaigul W, Priprem A, Limsitthichaikoon S (2021). Efficacy and safety of two artificial saliva-based polymers containing 0.1% pilocarpine for treatment of xerostomia: a randomized clinical pilot trial. J Clin Exp Dent.

[2] Soares MSM, Cavalcanti RL, Goncalves LFF, de Assis IO. Oral and systemic factors in xerostomia. RGO - Rev Gaúcha Odontol. 2021:69. 10.1590/1981-863720200003720200071.

[3] Jafari A, Alaee A, Ghods K (2021). The etiologies and considerations of dysgeusia: a review of literature. J Oral Biosci.

[4] Lexomboon D, Tan EC, Höijer J (2018). The effect of Xerostomic medication on oral health in persons with dementia. J Am Med Dir Assoc.

[5] Baharvand M, Khodadoustan A, Mohammadi M (2014). Xerostomia due to systemic disease: a review of 20 conditions and mechanisms. Ann Med Health Sci Res.

[6] Kapourani A, Kontogiannopoulos KN, Manioudaki A-E (2022). A review on xerostomia and its various management strategies: the role of advanced polymeric materials in the treatment approaches. Polymers (Basel).

[7] Millsop JW, Wang EA, Fazel N (2017). Etiology, evaluation, and management of xerostomia. Clin Dermatol.

[8] Fantozzi PJ, Pampena E, Di Vanna D, et al. Xerostomia, gustatory and olfactory dysfunctions in patients with COVID-19. Am J Otolaryngol. 2020;41(6). 10.1016/J.AMJOTO.2020.102721. 10.1016/j.amjoto.2020.102721 PMC7482593 32977063

[9] Liu B, Dion MR, Jurasic MM, Gibson G, Jones JA (2012). Xerostomia and salivary hypofunction in vulnerable elders: prevalence and etiology. Oral Surg Oral Med Oral Pathol Oral Radiol.

[10] Åstrøm AN, Lie SA, Ekback G, Gülcan F, Ordell S (2019). Self-reported dry mouth among ageing people: a longitudinal, cross-national study. Eur J Oral Sci.

[11] Davies AN, Thompson J. Parasympathomimetic drugs for the treatment of salivary gland dysfunction due to radiotherapy. Cochrane Database Syst Rev. 2015;2015(10):7-9. 10.1002/14651858.CD003782.pub3 PMC6599847 26436597

[12] Meshkatsadat M, Alaee A, Alirezaei S (2021). Management of Xerostomia in Covid-19 patints: a review article. Res Dent Sci.

[13] Chamani G, Zarei MR, Mehrabani M (2017). Assessment of systemic effects of ginger on salivation in patients with post-radiotherapy xerostomia. J Oral Heal Oral Epidemiol.

[14] Yang WF, Liao GQ, Hakim SG, Ouyang DQ, Ringash J, Su YX (2016). Is pilocarpine effective in preventing radiation-induced xerostomia? A systematic review and meta-analysis. Int J Radiat Oncol Biol Phys.

[15] Pronin AN, Wang Q, Slepak VZ (2017). Teaching an old drug new tricks: Agonism, antagonism, and biased signaling of pilocarpine through M3 muscarinic acetylcholine receptor. Mol Pharmacol.

[16] Little JW, Miller CS, Rhodus NL (2018). Little and Falace’s dental Management of the Medically Compromised Patient..

[17] Song J-I, Park J-E, Kim H-K, Kim M-E, Kim K-S (2017). Dose- and time-related effects of pilocarpine mouthwash on salivation. J Oral Med Pain.

[18] Malallah OS, Garcia CMA, Proctor GB, Forbes B, Royall PG (2018). Buccal drug delivery technologies for patient-centred treatment of radiation-induced xerostomia (dry mouth). Int J Pharm.

[19] Aromdee C, Ferguson MM, Ledger R, Wall J (1999). A pilot study of the disposition of pilocarpine in plasma, saliva and urine after a single oral dose. Eur J Pharm Sci.

[20] Bernardi R, Perin C, Becker FL (2002). Effect of pilocarpine mouthwash on salivary flow. Braz J Med Biol Res = Rev Bras Pesqui medicas e Biol.

[21] Adejoke HT, Louis H, Amusan OO, Apebende G (2019). A review on classes, extraction, purification and pharmaceutical importance of plants alkaloid. J Med Chem Sci.

[22] Nakamura N, Sasano N, Yamashita H (2009). Oral pilocarpine (5mg t.i.d.) used for xerostomia causes adverse effects in Japanese. Auris Nasus Larynx.

[23] de Pereira RMS, MDR B, Ferreira MP (2020). Topical pilocarpine for xerostomia in patients with head and neck cancer treated with radiotherapy. Oral Dis.

[24] Watanabe M, Yamada C, Komagata Y, Kikuchi H, Hosono H, Itagaki F. New low-dose liquid pilocarpine formulation for treating dry mouth in Sjögren’s syndrome: clinical efficacy, symptom relief, and improvement in quality of life. J Pharm Heal care Sci. 2018;4(1). 10.1186/S40780-018-0099-X. 10.1186/s40780-018-0099-x PMC5831212 29507747

[25] Park J-E, Song C-W, Kim K-S, Kim M-E (2015). Comparison of the effects of pilocarpine solution and tablet on salivary flow rate. J Oral Med Pain.

[26] Villa A, Connell CL, Abati S (2015). Diagnosis and management of xerostomia and hyposalivation. Ther Clin Risk Manag.

[27] Fox PC, Busch KA, Baum BJ (1987). Subjective reports of xerostomia and objective measures of salivary gland performance. J Am Dent Assoc.

[28] Karimi M (2019). Comparison of the efficacy of plaque removal of Listerine smart rinse kids and vi– one junior fluoridated mouthwash in children aged 6 to 10 years. Interv Pediatr Dent Open Access J.

[29] Duś-Ilnicka I, Krala E, Cholewińska P, Radwan-Oczko M. The use of saliva as a biosample in the light of covid-19. Diagnostics. 2021;11(10). 10.3390/diagnostics11101769. 10.3390/diagnostics11101769 PMC8534561 34679467

[30] Azizi A, Lavaf S, Najafi M (2007). Comparison of the effect of different chewing gums and one mouth wash on the amount and pH of saliva in healthy individuals. J Dent (Shiraz Univ Med Sci).

[31] Theunissen M, Rideaux-Seferina S, Magdelijns FJ, Janssen DJAA, van den Beuken-van EMHJJ (2021). Local Oral pilocarpine drops for relieving xerostomia (dry mouth) in the elderly: a pilot study. Vol 22.

[32] Zimmerman RP, Mark RJ, Tran LM, Juillard GF (1997). Concomitant pilocarpine during head and neck irradiation is associated with decreased posttreatment xerostomia. Int J Radiat Oncol Biol Phys.

[33] Nikles J, Mitchell GK, Hardy J (2015). Testing pilocarpine drops for dry mouth in advanced cancer using n-of-1 trials: a feasibility study. Palliat Med.

[34] Kim JH, Ahn HJ, Choi JH, Jung DW, Kwon JS (2014). Effect of 0.1% pilocarpine mouthwash on xerostomia: double-blind, randomised controlled trial. J Oral Rehabil.

[35] Turner MD (2016). Hyposalivation and xerostomia: etiology, complications, and medical management. Dent Clin N Am.

